# Prognostic value of metabolic tumor volume and total lesion glycolysis on preoperative ^18^F-FDG PET/CT in patients with localized primary gastrointestinal stromal tumors

**DOI:** 10.1186/s40170-021-00244-x

**Published:** 2021-01-28

**Authors:** Sang Hyun Hwang, Minkyu Jung, Yong Hyu Jeong, KwanHyeong Jo, Soyoung Kim, Jiyoung Wang, Arthur Cho

**Affiliations:** 1 Department of Nuclear Medicine, Severance Hospital, Yonsei University College of Medicine, 50-1 Yonsei-ro, Seodaemun-gu, Seoul, 03722 Republic of Korea; 2 Division of Medical Oncology, Department of Internal Medicine, Yonsei Cancer Center, Yonsei University College of Medicine, Seoul, Republic of Korea; 3 Department of Nuclear Medicine, Yongin Severance Hospital, Yonsei University College of Medicine, Seoul, Republic of Korea; 4 Department of Nuclear Medicine, Korea University Anam Hospital, Korea University College of Medicine, Seoul, Republic of Korea; 5 Department of Nuclear Medicine, Gangnam Severance Hospital, Yonsei University College of Medicine, Seoul, Republic of Korea

**Keywords:** Gastrointestinal stromal tumor, ^18^F-Fluorodeoxyglucose, PET, Metabolic tumor volume, Prognosis

## Abstract

**Background:**

This study aimed to evaluate the prognostic value of pretreatment ^18^F-fluorodeoxyglucose positron emission tomography/computed tomography (^18^F-FDG PET/CT) in patients with localized primary gastrointestinal stromal tumors (GISTs) and to compare the predictive values of ^18^F-FDG PET/CT parameters with those of clinicopathological prognostic factors.

**Methods:**

Sixty-two localized GIST patients who underwent staging with ^18^F-FDG PET/CT from January 2007 to December 2013 before surgery were retrospectively enrolled. A volume of interest with a standardized uptake value (SUV) threshold of 2.5 was used to determine the metabolic tumor volume (MTV) and total lesion glycolysis (TLG). These metabolic indices, along with the maximum SUV (SUVmax), were analyzed to evaluate recurrence-free survival (RFS). Other significant clinical and pathologic indices were also retrospectively reviewed for RFS analysis.

**Results:**

Patients were followed up for a median of 42.0 months (range, 5.6–111.5). During the follow-up period, 13 patients (21.0%) experienced disease recurrence. In univariate analysis, tumor size (> 5 cm), mitotic count (> 5/high-power field), modified National Institutes of Health (NIH) consensus criteria, adjuvant imatinib treatment, SUVmax (≥ 7.04), MTV (≥ 50.76 cm^3^), and TLG (≥ 228.79 g) were significant prognostic factors affecting RFS (*p* < 0.05). In multivariate analysis, only MTV (hazard ratio, 17.69; 95% confidence interval [CI], 2.03–154.17, *p* = 0.009) and TLG (hazard ratio, 20.48; 95% CI, 2.19–191.16, *p* = 0.008) were independent prognostic factors for RFS. The 5-year RFS rates were 96.4% and 96.6% in patients with a low MTV and TLG and 27.3% and 23.6% in patients with a high MTV and TLG, respectively (*p* < 0.001).

**Conclusion:**

MTV and TLG are independent prognostic factors for predicting recurrence in patients with localized primary GIST. Patients with a high MTV or TLG are at risk for poor prognosis and should be closely observed for disease recurrence.

**Supplementary Information:**

The online version contains supplementary material available at 10.1186/s40170-021-00244-x.

## Background

Gastrointestinal stromal tumors (GISTs) are the most common mesenchymal tumors of the gastrointestinal tract, of which the stomach and small intestine are the most common locations [1]. As GISTs present with a wide spectrum of benign to malignant findings, all GISTs are considered to have malignant potential [2], and surgery is the standard treatment option for localized primary GIST for curative intent [3]. However, tumor recurrence is common in the original tumor site, liver, or peritoneum, with a recurrence rate of 50% within 5 years [4]. Due to KIT proto-oncogene or platelet-derived growth factor receptor α (*PDGFRα*) mutations in GIST [5, 6], targeted therapy using imatinib mesylate (Gleevec®, Novartis Pharmaceuticals, Basel, Switzerland), a selective inhibitor of KIT and PDGFRα proteins, has been shown to prolong recurrence-free survival (RFS) in those at high risk for recurrence when used in an adjuvant therapy setting [7]. Current guidelines for risk stratification of GIST are based on tumor size and mitotic count, primary tumor location, and tumor rupture [8–10], most of which are assessed based on pathologic specimens.

^18^F-Fluorodeoxyglucose positron emission tomography/computed tomography (^18^F-FDG PET/CT) has been reported to be a useful imaging method for staging and for monitoring responses to adjuvant imatinib therapy in GIST [11–15]. Moreover, ^18^F-FDG PET/CT can be used to non-invasively evaluate tumor glycolysis, which is correlated with mitotic count in many tumors, including GIST [16]. As such, it is possible to directly compare current guidelines for GIST with preoperative ^18^F-FDG PET/CT findings in terms of predicting RFS. Potentially, ^18^F-FDG PET/CT findings may be better at predicting RFS than the current guidelines, as global tumor glycolysis is readily assessed using volumetric parameters, such as metabolic tumor volume (MTV) or total lesion glycolysis (TLG), in contrast to mitotic count, which has been shown to be inhomogeneous in larger GISTs [17, 18]. However, only a few studies to date have evaluated the prognostic value of the FDG uptake pattern on PET/CT [19, 20]. Additionally, ^18^F-FDG PET/CT may be helpful in guiding pathologists in the identification of areas with high glycolysis, as these areas may have a higher Ki-67 expression.

In this study, we evaluated the prognostic value of these volumetric parameters on preoperative ^18^F-FDG PET/CT in patients with localized primary GIST who underwent curative resection and compared their predictive values with those of clinicopathological prognostic factors.

## Methods

### Patients

The institutional review board of our university approved this retrospective study, and the requirement to obtain informed consent was waived (IRB approved no. 4-2016-0914). We retrospectively reviewed electronic medical records of localized primary GIST patients who underwent preoperative ^18^F-FDG PET/CT between January 2007 and December 2013. Of these patients, 62 were enrolled in the present study. The patients who had a history of any other malignancy, who had unresectable cancer on imaging studies, who had distant metastasis, or who had received neoadjuvant treatment before surgery were excluded from this study. The median interval between preoperative ^18^F-FDG PET/CT and surgery was 11 days (range 1–74 days). All patients underwent post-surgical clinical follow-up every 3–6 months, including contrast-enhanced CT scan according to their clinical condition.

### ^18^F-FDG PET/CT scan

All patients underwent ^18^F-FDG PET/CT scan using either a Biograph 40 TruePoint PET/CT scanner (Siemens Healthcare, Erlangen, Germany) or Discovery STe PET/CT scanner (GE Healthcare, Waukesha, WI, USA). The patients fasted for at least 6 h, and glucose levels in the peripheral blood were confirmed to be lower than 140 mg/dL before ^18^F-FDG injection. Approximately 5.5 MBq of ^18^F-FDG per kilogram of body weight was administered intravenously 1 h before image acquisition. After the initial low-dose CT (Biograph 40 TruePoint, 36 mA, 120 kVp; Discovery STe, 30 mA, 130 kVp) without contrast-enhancement, standard PET imaging from the neck to the proximal thighs with an acquisition time of 2.5 min/bed position in 3-dimensional mode was performed. The PET images were reconstructed using ordered-subset expectation maximization (2 iterations, 20 subsets).

### Image analysis

All ^18^F-FDG PET/CT images were reviewed by two nuclear medicine physicians, and discrepancies between the readers were resolved by a consensus reading. The location of GIST lesions on PET/CT and contrast-enhanced CT images was decided using a fusion module in MIM version 6.5 (MIM Software Inc., Cleveland, OH, USA). The maximum standardized uptake value (SUVmax) and MTV were measured in a volume of interest (VOI) drawn on PET images. The SUVmax of the VOI was measured as (decay-corrected activity [kBq] per tissue volume [mL])/(injected ^18^F-FDG activity [kBq] per body mass [g]). MTV was defined as total tumor volume with an SUV of ≥ 2.5, and the MTV and SUVmean of the VOI were automatically calculated. TLG was calculated as SUVmean × MTV.

In addition, we visually analyzed the GIST uptake patterns on PET images, according to the criteria suggested by Miyake et al. [19].

### Statistical analysis

The following variables were included in the statistical analysis: age, sex, tumor site, tumor size, mitotic count per high-power field (HPF), resection, adjuvant imatinib treatment, ^18^F-FDG PET/CT parameters (SUVmax, MTV, and TLG), and the modified National Institutes of Health (NIH) consensus criteria. For the statistical analysis, all continuous variables were divided into two groups. The specific cut-off values for ^18^F-FDG PET/CT parameters were determined using the Contal and O’Quigley method [21], and the modified NIH consensus criteria were categorized into high-risk and other risk groups [22]. Spearman’s correlation analysis was used to evaluate the relationships between tumor size and ^18^F-FDG PET/CT parameters. Kruskal–Wallis test and Dunn’s post hoc analysis were performed to compare ^18^F-FDG PET/CT parameters among the mitotic count groups. The Kruskal–Wallis test and Dunn’s post hoc analysis were also performed to evaluate the results of visual analysis of the MTV.

Survival was calculated from the date of surgical resection to the date of recurrence or the last follow-up visit at our hospital. The predictive significance of the evaluated variables was evaluated using the Cox proportional hazards regression test for univariate and multivariate analyses. Parameters with *p* values < 0.05 in the univariate analysis were included in the multivariate analysis. Multicollinearity among SUVmax, MTV, and TLG was evaluated by calculating the Spearman rank correlation coefficients prior to the multivariate analysis. A Kaplan–Meier survival analysis was performed to calculate cumulative RFS, and the results were compared using the log-rank test.

Time-dependent receiver operating characteristics (ROC) curves were used to evaluate the performance of PET parameters and the modified NIH consensus criteria in relation to the accuracy of prediction of the risk of tumor recurrence. We compared the global concordance probability (integrated area under the curve, or iAUC) of each variable adjusted by adjuvant imatinib treatment [23]. The iAUC is a weighted average of the AUC during a follow-up period, and a larger iAUC corresponds to a better predictive accuracy. Differences between PET parameters and modified NIH consensus criteria were evaluated by bootstrapping with resampling 1000 times.

All statistical analyses were conducted using SAS version 9.4 (SAS Inc., Cary, NC, USA) and R version 3.1.3 (http://www.R-project.org). *p* values less than 0.05 were considered statistically significant.

## Results

### Patients’ characteristics

The characteristics of all enrolled patients are shown in Table 1. Patients were distributed according to the modified NIH consensus criteria as follows: very low risk (*n* = 1; 1.6%), low risk (*n* = 30; 48.4%), intermediate risk (*n* = 6; 9.7%), and high risk (*n* = 25; 40.3%). Fifty-nine patients (95.2%) underwent microscopic radical resection (R0), and 14 (22.6%) received adjuvant imatinib treatment after surgery. There were no patients with tumor rupture before and during surgery. The median duration of clinical follow-up was 42.0 months (range, 5.6–111.5 months). During the follow-up period, 13 patients (21.0%) experienced disease recurrence and three (4.8%) died.

**Table 1:** Patient characteristics (*n* = 62)

Characteristics	Number of patients (%)
Age (years)	Median 64 (range 20–93)
Sex	
Male	31 (50.0)
Female	31 (50.0)
Site of tumor	
Gastric	29 (46.8)
Non-gastric	33 (53.2)
Size (cm)	Median 5.0 (range 2.0–32.0)
Mitotic count per HPFs	
≤ 5	42 (67.7)
> 5 and ≤ 10	6 (9.7)
> 10	14 (22.6)
Modified NIH consensus criteria	
Very low	1 (1.6)
Low	30 (48.4)
Intermediate	6 (9.7)
High	25 (40.3)
Resection	
R0	59 (95.2)
R1	3 (4.8)
Adjuvant imatinib treatment	
Yes	14 (22.6)
No	48 (77.4)
PET metabolic indices	
SUVmax	Median 4.18 (range 1.39–27.56)
MTV (cm^3^)	Median 9.57 (range 0.00–1559.19)
TLG (g)	Median 29.44 (range 0.00–8770.89)

*HPF* high-power field, *NIH* National Institutes of Health, *SUV* standard uptake value, *MTV* metabolic tumor volume, *TLG* total lesion glycolysis

### Correlation of ^18^F-FDG PET/CT parameters with pathology

Tumor size showed a moderate correlation with PET-derived tumor volumes (MTV: *ρ* = 0.656, TLG: *ρ* = 0.638, *p* < 0.001 for each) and a weak correlation with SUVmax (*ρ* = 0.492, *p* < 0.001), supporting the notion that single dimensional measurements of pathologic size provide good estimations of tumor volume. Conversely, the difference in tumor size and MTV or TLG may be attributed to the inaccuracies of metabolism-derived tumor volume measurement, as our definition of tumor volume only included tumor volumes with an SUV of > 2.5. Statistical analysis of visual analysis of FDG distribution with MTV revealed that a ring-shaped pattern was more often seen in tumors with a larger MTV compared to homo/diffuse or unclassified pattern (Supplemental Table 1).

Mitotic count was classified as follows: ≤ 5/50 per HPFs, > 5 and ≤ 10/50 per HPFs, and > 10/50 per HPFs. There was a significant correlation between stratified mitotic counts and PET-derived metabolomic measurements. Tumors with a higher mitotic count had a higher SUVmax, MTV, and TLG (*p* < 0.001). Dunn’s post hoc test showed that compared with tumors with a mitotic count ≤ 5/50 per HPFs, tumors with a mitotic count > 10/50 had a significantly high SUVmax (3.55, interquartile range [IQR] 2.53–5.16 vs. 10.69, IQR 4.55–14.94, *p* < 0.001), MTV (4.38 cm^3^, IQR 0.15–12.77 cm^3^ vs. 98.88 cm^3^, IQR 22.98–581.05 cm^3^, *p* < 0.001), and TLG (12.20 g, IQR 0.36–43.63 g vs. 600.10 g, IQR 105.46–3082.78 g, *p* < 0.001). Post hoc test also showed that comparison between the mitotic count ≤ 5/50 and > 5 and ≤ 10/50 groups revealed that only MTV and TLG were significantly higher in the mitotic count > 5 and ≤ 10/50 per HPFs groups (86.64 cm^3^ [IQR 20.60–129.23 cm^3^, *p* = 0.001] and 345.83 g [IQR 77.59–457.18 g, *p* = 0.001]); SUVmax was only borderline significantly higher (5.91, IQR4.11–11.12, *p* = 0.025).

### Univariate and multivariate survival analyses

The optimal cut-off values for SUVmax, MTV, and TLG were 7.04, 54.76 cm^3^, and 228.79 g, respectively, as determined by the Contal and O’Quigley method. The significant values of variables for predicting RFS in univariate and multivariate analyses are shown in Table 2. Tumor size, mitotic count per HPFs, the modified NIH consensus criteria, adjuvant imatinib treatment, SUVmax, MTV, and TLG were significant prognostic factors in univariate analyses (*p* = 0.042, 0.016, 0.019, 0.005, 0.001, < 0.001, and < 0.001, respectively). Tumor size and mitotic count were excluded from multivariate analyses, as these factors are included in the modified NIH consensus criteria. Although the visual analysis was significant as the prognostic factor in univariate analysis, it was not significant in the multivariate analysis (Supplemental Table 2). Since there was a significant correlation between MTV and TLG (*r* = 0.996, *p* < 0.001), MTV and TLG were assessed separately. In the multivariate analyses, only MTV (*p* = 0.009; hazard ratio, 17.69; 95% CI, 2.03–154.17) and TLG (*p* = 0.008; hazard ratio, 20.48; 95% CI, 2.19–191.16) were highlighted as independent prognostic factors for RFS.

**Table 2:** Univariate and multivariate analyses of recurrence-free survival (*n* = 62)

Variable	Univariate analysis		Multivariate analysis (MTV model)		Multivariate analysis (TLG model)	
	Hazard ratio (95% CI)	*p* value	Hazard ratio (95% CI)	*p* value	Hazard ratio (95% CI)	*p* value
Age (> 60 years vs. ≤ 60 years)	1.88 (0.57–6.19)	0.298				
Sex (men vs. women)	0.62 (0.21–1.85)	0.389				
Site of tumor (gastric vs. non-gastric)	1.19 (0.40–3.57)	0.752				
Size (> 5 cm vs. ≤ 5 cm)	3.83 (1.05–13.98)	0.042*				
Mitotic count per HPFs (> 5 vs. ≤ 5)	4.27 (1.31–13.89)	0.016*				
Resection (R1 vs. R0)	2.48 (0.32–19.45)	0.389				
Modified NIH consensus criteria (high-risk group vs. the other risk groups)	4.67 (1.28–16.98)	0.019*	0.34 (0.05–2.26)	0.261	0.33 (0.048–2.24)	0.255
Adjuvant imatinib treatment (yes vs. no)	4.89 (1.62–14.77)	0.005*	1.28 (0.34–4.77)	0.715	1.39 (0.36–5.35)	0.632
SUVmax (≥ 7.04 vs. < 7.04)	13.46 (2.98–60.75)	0.001*	4.44 (0.79–25.00)	0.091	3.43 (0.53–22.17)	0.196
MTV (≥ 54.76 cm^3^ vs. < 54.76 cm^3^)	19.79 (4.37–89.67)	< 0.001*	17.69 (2.03–154.17)	0.009*		
TLG (≥ 228.79 g vs. < 228.79 g)	22.24 (4.91–100.77)	< 0.001*			20.48 (2.19–191.16)	0.008*

*HPF* high-power field, *SUV* standard uptake value, *MTV* metabolic tumor volume, *TLG* total lesion glycolysis, *CI* confidence interval

*Bold *p* value: statistically significant (*p* < 0.05)

### Kaplan–Meier analyses of recurrence-free survival

**Fig. 1:**
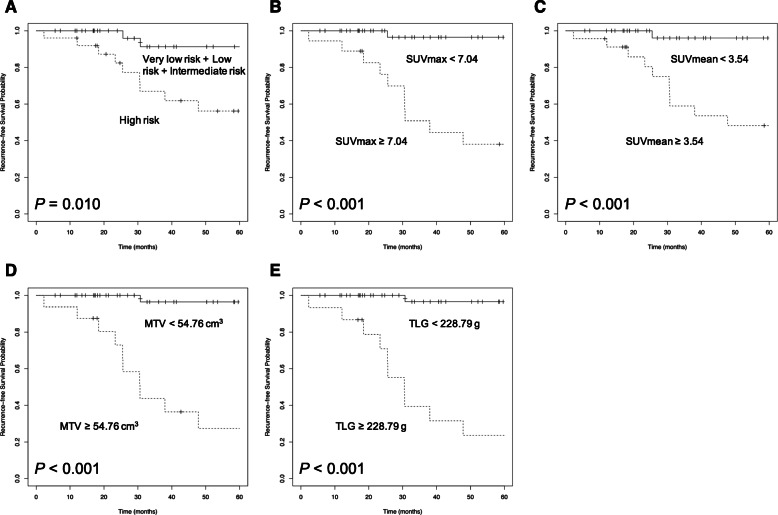
Cumulative recurrence-free survival curves of the enrolled patients (*n* = 62). According to the modified National Institutes of Health (NIH) consensus criteria (**a**), maximum standard uptake value (SUVmax) (**b**), mean standard uptake value (SUVmean) (**c**), metabolic tumor volume (MTV) (**d**), and total lesion glycolysis (TLG) (**e**)

According to the modified NIH consensus criteria, the 5-year RFS rate was 91.3% in patients with very low, low, and intermediate risk, compared with 56.2% in patients at high risk (*p* = 0.010, Fig. 1a). The 5-year RFS rate was 96.4% in patients with a low SUVmax, compared with 38.1% in patients with a high SUVmax (*p* < 0.001, Fig. 1b). Similarly, the 5-year RFS rate was higher in patients with a low SUVmean than in those with a high SUVmean (96.0% vs. 48.2%, *p* = 0.001, Fig. 1c). The 5-year RFS rate was 96.4% in patients with a low MTV, compared with 27.3% in patients with a high MTV (*p* < 0.001, Fig. 1d). There was also a statistically significant difference in 5-year RFS rates with respect to TLG (96.6% for low TLG vs. 23.6% for high TLG patients; *p* < 0.001, Fig. 1e). The visual analysis of FDG uptake patterns revealed a lower 5-year RFS rate in patients with the ring-shaped pattern than in those with the non-ring-shaped pattern (50.9% vs. 82.3%, *p* = 0.003, Supplemental Fig. 1).

### Assessment of predictive performance

**Fig. 2:**
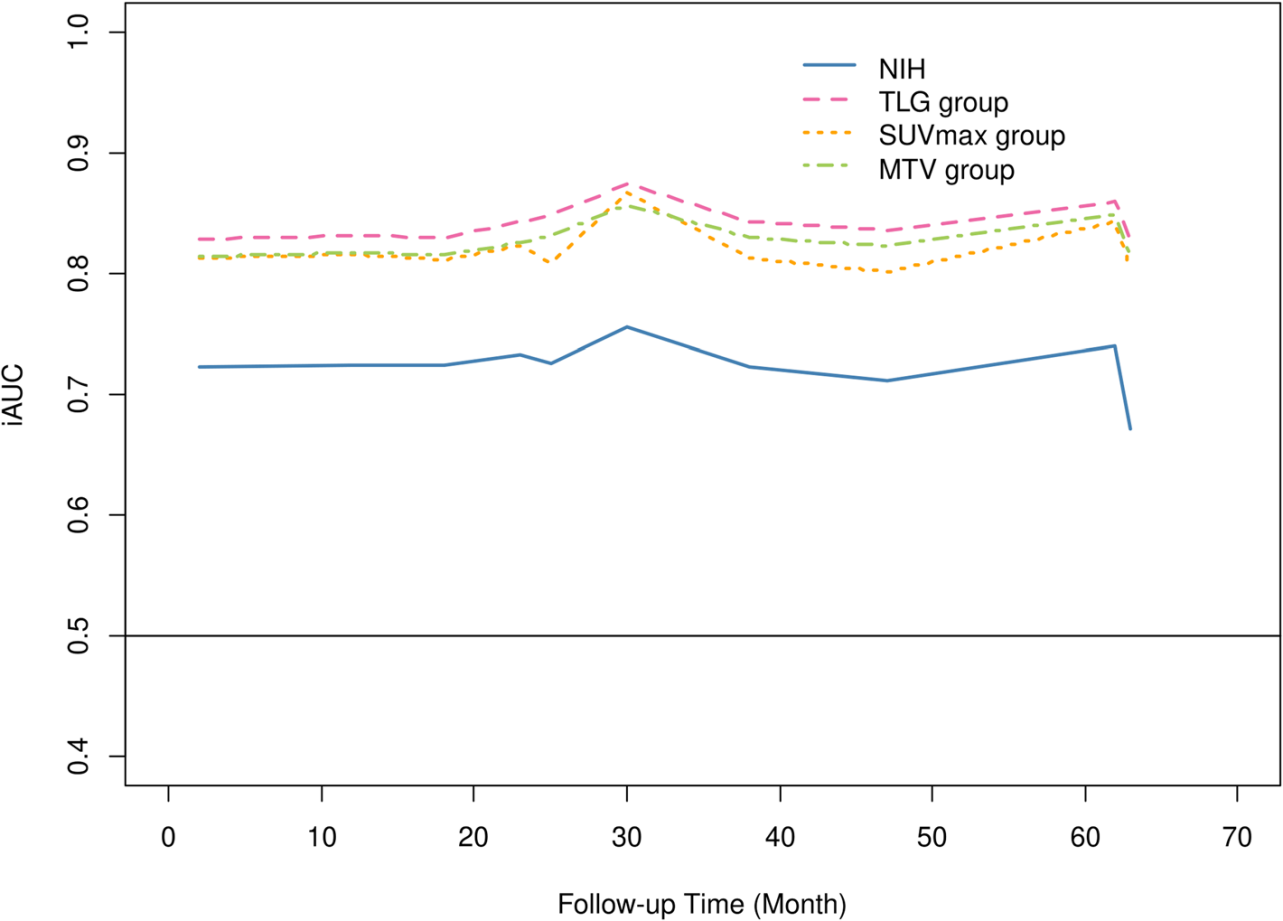
Time-dependent ROC curve analysis for predicting recurrence-free survival in patients with localized primary GIST. According to the modified NIH criteria, SUVmax, MTV, and TLG. All variables are adjusted with regard to adjuvant imatinib treatment. SUV, standard uptake value; MTV, metabolic tumor volume; TLG, total lesion glycolysis; ROC, receiver operating characteristics

**Fig. 3:**
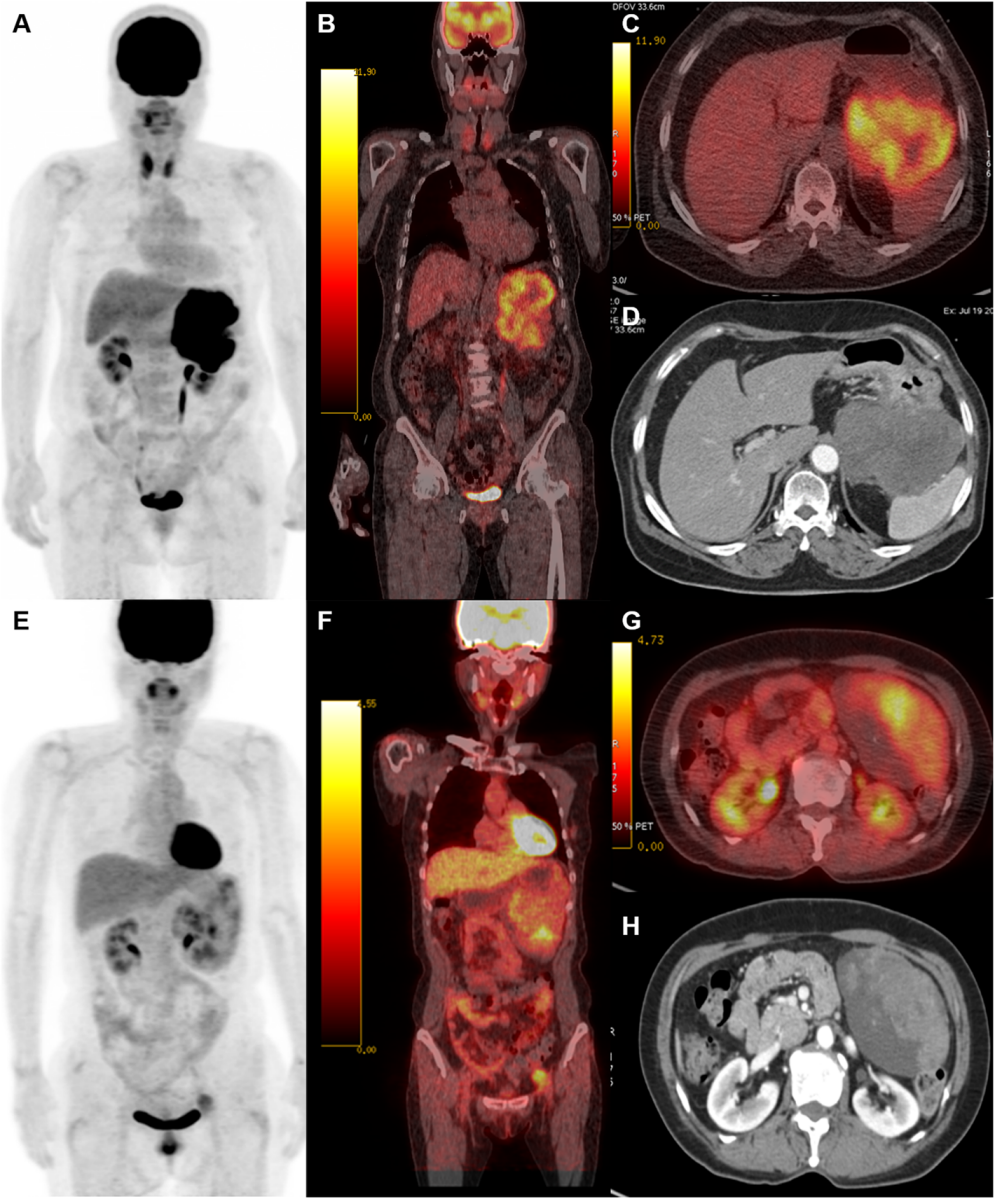
Representative cases. Patients with malignant gastrointestinal stromal tumors (GISTs) of similar size and mitotic count but different total lesion glycolysis (TLG) values and prognosis. **a**–**d** A 65-year-old woman with gastric GIST (size 13 cm, > 10/50 per high-power field (HPF) mitotic count, high-risk National Institutes of Health [NIH] criteria). The maximum standard uptake value (SUVmax) was 12.99, metabolic tumor volume (MTV) was 699.64 cm^3^, and TLG was 4350.31 g. Tumor recurrence was noted at 30.6 months after surgery. **e**–**h** A 68-year-old woman with gastric GIST (size 12.3 cm, > 10/50 per HPFs mitotic count, high-risk NIH criteria). SUVmax 4.62, MTV 76.75 cm^3^, TLG 221.67 g. This patient had no evidence of recurrence (42.7 months of follow-up after surgery). **a**, **e** ^18^F-Fluorodeoxyglucose positron emission tomography (^18^F-FDG PET) maximum intensity projection. **b**, **f** Coronal fusion ^18^F-FDG PET/computed tomography (CT). **c**, **g** Transaxial fusion ^18^F-PET/CT. **d**, **h** Transaxial contrast-enhanced CT. Intensity of ^18^F-FDG uptake in fusion images **b** and **c** were adjusted to show intratumoral inhomogeneous uptake

Multivariate time-dependent ROC curve analysis during the follow-up period is presented in Fig. 2. Values for iAUC for the modified NIH consensus criteria, SUVmax, MTV, and TLG were 0.76 (95% CI, 0.63–0.88), 0.86 (95% CI, 0.77–0.93), 0.87 (95% CI, 0.77–0.93), and 0.89 (95% CI, 0.80–0.95), respectively. In the model for iAUC comparison between the modified NIH consensus criteria and each ^18^F-FDG PET/CT parameter, there were no significant differences between the modified NIH criteria and the SUVmax (0.10, 95% CI 0–0.23); however, the iAUC of the MTV was significantly higher than those of the modified NIH criteria (0.11, 95% CI 0.02–0.24) and TLG (0.13, 95% CI 0.03–0.25). Volumetric parameters of ^18^F-FDG PET/CT (MTV and TLG) showed better predictive accuracy than the modified NIH consensus criteria (Fig. 2, representative cases are shown in Fig. 3).

## Discussion

In this study, we evaluated the utility of ^18^F-FDG volumetric parameters for predicting prognosis in patients with GISTs. One often used clinical assessment for the risk of GIST recurrence is the modified NIH consensus criteria, which determines the risk of recurrence based on tumor size and mitotic count of the tumor [10]. Because mitotic activity is correlated with tumor growth, it is possible that mitotic count is correlated with tumor glycolysis, a major metabolic pathway needed for rapid tumor growth [24]. In this regard, studies have shown that ^18^F-FDG uptake reflects tumor glycolysis in situ, which may suggest that mitotic count is likely to be correlated with ^18^F-FDG uptake in tumors [16, 25]. Tumor volume may also be easily and reliably measured in situ based on ^18^F-FDG uptake, as MTV and TLG are well-established methodologies in measuring tumor volume [26, 27]. There are several methods to measure MTV and TLG, such as fixed absolute threshold-based methods, fixed relative threshold-based methods, and algorithm-based methods. In this study, a fixed threshold of SUV 2.5 was used since it has shown good predictive value for prognosis, has shown the best inter-observer agreement, and is easily measured in clinical settings. The limitation of this method is that tumors with an ^18^F-FDG uptake lower than the fixed absolute threshold may be excluded from MTV and TLG measurements [27–29] or their measurements may not accurately reflect the pathologic size of the specimen. These two factors, MTV or TLG, and ^18^F-FDG uptake in GIST may correlate with the modified NIH consensus criteria and may potentially predict patient prognosis better than the NIH classification does, as these pathology-based assessments may not reflect the total tumor volume or mitotic count in the whole tumors, as intratumoral sampling bias may occur [17, 18]. Therefore, volumetric parameters, such as TLG, have the advantage of representing tumor metabolism as a whole without the potential for selection bias.

We have shown in our study that there is a moderate correlation between pathologic tumor size and image-based tumor volumetry. One likely reason for the moderate correlation is the measurement error inherent to metabolism-based imaging thresholding; another potential factor may be that one-dimensional pathologic assessment of tumor size may not accurately measure tumor volume. We have also shown that ^18^F-FDG uptake reflects mitotic count, as tumors with moderate to high mitotic count showed higher ^18^F-FDG uptake than tumors with low mitotic count. Based on these results, we proceeded to evaluate the prognostic ability of ^18^F-FDG PET/CT in predicting patient pathology, as TLG may reflect both mitotic count and tumor size and it importantly combines these factors into a single quantifiable number [30]. We have shown that the MTV and TLG of preoperative GIST lesions are independent prognostic factors for predicting RFS and that they predict survival more accurately than the modified NIH criteria. Although the SUVmax of preoperative GIST lesions showed no significant difference in predicting survival compared to the modified NIH criteria, a larger cohort could result in a statistically significant value. Although MTV and TLG showed significantly higher predictive ability compared to the NIH criteria in predicting RFS, the imaging metrics highly overlap, which is suggestive of the robustness of this methodology in evaluating patient prognosis. Further studies with a larger sample size are needed to evaluate the accuracy of prediction of the risk of tumor recurrence.

The standard treatment protocol of localized GIST is complete surgical resection, and patients with a significant risk of recurrence undergo adjuvant imatinib treatment [3]. Proper selection of patients who are at high risk is important, as a randomized trial demonstrated that high-risk patients require 3 years of adjuvant treatment rather than 1-year-long treatment to show RFS improvement [31]. However, prognostic factors, such as mitotic count, exact tumor size, tumor rupture during surgery, and surgical resection margin, can only be assessed postoperatively. In this regard, non-invasive imaging modalities and volumetric parameters of ^18^F-FDG PET/CT may help in predicting prognosis before treatment and potentially guide pathologists in locating areas that might have high Ki-67 values, which may result in more accurate assessment of patient prognosis using the NIH criteria. Further studies are needed to evaluate the possible role of FDG PET/CT in pathologic assessment.

To date, two studies have evaluated the significance of preoperative ^18^F-FDG PET/CT for predicting prognosis in patients with localized primary GIST [19, 20]. Miyake et al. categorized ^18^F-FDG uptake patterns as ring-shaped, homogenous/diffuse, heterogeneous/partial, or unclassified and showed that ring-shaped uptake on preoperative ^18^F-FDG PET/CT was a significant prognostic factor for localized primary GISTs, which we have confirmed in our studies. However, they did not evaluate quantitative parameters, such as SUV, MTV, or TLG, and we found that this visual analysis was not significant in multivariate analysis. Albano et al. showed that preoperative MTV and TLG were independent prognostic factors for localized primary GIST. In addition to their findings, we have shown that metabolic ^18^F-FDG PET/CT parameters are strong prognostic factors for RFS.

Our study had several limitations. First, this was a retrospective single-center study, with a relatively small number of patients. Thus, selection bias might be inherent. This may also be the reason that SUVmax did not show better predictive accuracy than modified NIH criteria. Therefore, larger population studies are needed to confirm our results. We also used two different PET/CT scanners, which may have influenced the SUV measurements. Second, because most GISTs arise in the stomach and small intestine, physiologic ^18^F-FDG uptake in the stomach or small intestine could mask primary lesions or result in difficult tumor thresholding. However, using a fusion module provided by imaging software, ^18^F-FDG uptake by primary GISTs was evaluated carefully without including physiologic uptake. Third, although we have shown that mitotic count is correlated with ^18^F-FDG uptake, there are many other factors such as hypoxia, hyperemia, and necrosis that may influence ^18^F-FDG uptake. Further studies are needed to evaluate factors related to ^18^F-FDG uptake in GIST.

## Conclusions

Preoperative MTV and TLG have a high predictive prognostic value for RFS in patients with localized primary GIST. Patients with a high MTV or TLG on ^18^F-FDG PET/CT show shorter RFS and should be closely observed for recurrence.

## Supplementary Information

**Additional file 1: Supplemental Table 1.** Correlation analysis between visual FDG uptake pattern with metabolic tumor volume. **Supplemental Table 2.** Univariate and multivariate analyses of recurrence-free survival (*n*=62). **Supplementary Figure 1.** Cumulative recurrence-free survival curves according to ^18^F-FDG uptake patterns (*n*=62).

## Data Availability

Data will be provided upon reasonable request.

## Abbreviations

^18^F-FDG PET/CT: ^18^F-Fluorodeoxyglucose positron emission tomography/computed tomography
CI: Confidence interval
CT: Computed tomography
GISTs: Gastrointestinal stromal tumors
HPF: High-power field
iAUC: Integrated area under the curve
IQR: Interquartile range
MTV: Metabolic tumor volume
NIH: National Institutes of Health
PET: Positron emission tomography
RFS: Recurrence-free survival
ROC: Receiver operating characteristics
SUV: Standard uptake value
SUVmax: Maximum standard uptake value
SUVmean: Mean standard uptake value
TLG: Total lesion glycolysis
VOI: Volume of interest
