# Supplementary material for: Prognostic value of metabolic tumor volume and total lesion glycolysis on preoperative 18F-FDG PET/CT in patients with localized primary gastrointestinal stromal tumors
Source: Cancer Metab. 2021 Jan 28;9:8. doi: 10.1186/s40170-021-00244-x (PMC7844977; doi:10.1186/s40170-021-00244-x)

**Supplemental Table 1**. Correlation analysis between visual FDG uptake pattern with metabolic tumor volume

| FDG uptake pattern | MTV (cm^3^)  (median, IQR) | *P* value (vs. Ring-shaped) |
| --- | --- | --- |
| Ring-shaped (n=15) | 83.9 (12.4~541.6) |  |
| Homo/diffuse (n=24) | 1.0 (0.99~25.3) | 0.001 |
| Hetero/partial (n=14) | 18.5 (5.5~87.8) | 0.085 |
| Unclassified (n=9) | 0.0 (0.0~0.36) | <0.001 |

*Values are represented in median (25%~75% IQR)

** p-values are Dunn’s post hoc analysis

**Supplemental Table 2.** Univariate and multivariate analyses of recurrence-free survival (n=62)

| Variable | Univariate analysis | | Multivariate analysis  (MTV model) | | Multivariate analysis  (TLG model) | |
| --- | --- | --- | --- | --- | --- | --- |
|  | Hazard ratio  (95% CI) | *P* value | Hazard ratio  (95% CI) | *P* value | Hazard ratio  (95% CI) | *P* value |
| Age (>60 years vs. ≤60 years) | 1.88  (0.57-6.19) | 0.298 |  |  |  |  |
| Sex (Men vs. Women) | 0.62  (0.21-1.85) | 0.389 |  |  |  |  |
| Site of tumor (Gastric vs. Non-gastric) | 1.19  (0.40-3.57) | 0.752 |  |  |  |  |
| Size (>5 cm vs. ≤5 cm) | 3.83  (1.05-13.98) | **0.042*** |  |  |  |  |
| Mitotic count per HPFs (>5 vs. ≤5) | 4.27  (1.31-13.89) | **0.016*** |  |  |  |  |
| Resection (R1 vs. R0) | 2.48  (0.32-19.45) | 0.389 |  |  |  |  |
| Modified NIH consensus criteria (high risk group vs. the other risk groups) | 4.67  (1.28-16.98) | **0.019*** | 0.41  (0.06-2.81) | 0.362 | 0.38  (0.05-2.72) | 0.338 |
| Adjuvant imatinib treatment (Yes vs. No) | 4.89  (1.62-14.77) | **0.005*** | 1.13  (0.28-4.52) | 0.865 | 1.24  (0.30-5.15) | 0.772 |
| PET pattern (Ring-shaped vs. Not ring-shaped) | 4.61  (1.53-13.91) | **0.007*** | 1.63  (0.44-6.00) | 0.461 | 1.48  (0.40-5.58) | 0.555 |
| SUVmax (≥7.04 vs. <7.04) | 13.46  (2.98-60.75) | **0.001*** | 4.78  (0.81-28.16) | 0.084 | 3.89  (0.57-26.52) | 0.166 |
| MTV (≥54.76 cm^3^ vs. <54.76 cm^3^) | 19.79  (4.37-89.67) | **<0.001*** | 12.75  (1.29-126.14) | **0.029*** |  |  |
| TLG (≥228.79 g vs. < 228.79 g) | 22.24  (4.91-100.77) | **<0.001*** |  |  | 15.12  (1.37-167.44) | **0.027*** |

HPF, high-power field; PET, positron emission tomography; SUV, standard uptake value; MTV, metabolic tumor volume; TLG, total lesion glycolysis

Bold *P* value*: statistically significant (*P* < 0.05)

**Supplementary Figure 1.** Cumulative recurrence-free survival curves according to ^18^F-FDG uptake patterns (n=62).


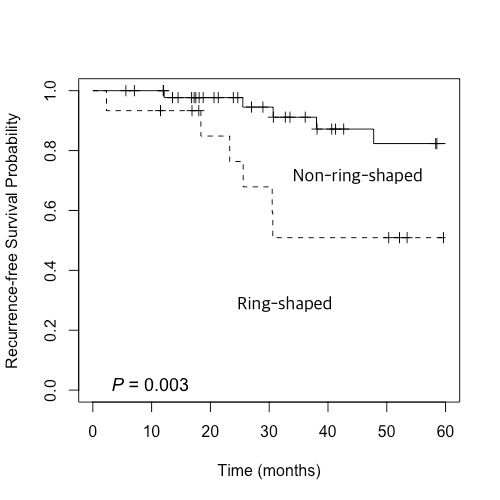

Supplement: Supplementary file 1 — Additional file 1: Supplemental Table 1. Correlation analysis between visual FDG uptake pattern with metabolic tumor volume. Supplemental Table 2. Univariate and multivariate analyses of recurrence-free survival (n=62). Supplementary Figure 1. Cumulative recurrence-free survival curves according to 18F-FDG uptake patterns (n=62). [file 40170_2021_244_MOESM1_ESM.docx]
